# Associations between weight concern, bullying, cigarette use, and vaping among heterosexual and sexual minority female adolescents

**DOI:** 10.1016/j.puhe.2026.106266

**Published:** 2026-04-02

**Authors:** J.P. Calzo, K. Quiballo, S.W. Kelsey, Y.B.A. Floresca, A. Shigemori, G. Phillips

**Affiliations:** a School of Public Health, San Diego State University, San Diego, CA, USA; b Action Research on Community Health Equity and Stigma Lab, Institute for Behavioral and Community Health, San Diego, CA, USA; c Feinberg School of Medicine, Northwestern University, USA

**Keywords:** Cigarette smoking, Vaping, Sexuality, Bullying, Body image

## Abstract

**Objective:**

The objective of this study was to evaluate the influence of weight concern and bullying on sexual orientation disparities in cigarette use and vaping among adolescent females.

**Study design:**

Cross-sectional analysis.

**Methods:**

Using pooled data from 2009 to 2019 Youth Risk Behavior Surveys (n = 233,437), multivariable logistic regression models examined cross-sectional associations between weight perception and change efforts, bullying, and smoking or vaping behaviors.

**Results:**

Relative to heterosexual adolescent females, bisexual females had elevated odds of initiating smoking before age 13 (AOR = 1.57, 95% CI = 1.08–2.28), while lesbian (AOR = 3.92, 95% CI = 2.07–7.42) and bisexual (AOR = 3.39, 95% CI = 2.39–4.81) females reported elevated odds of current smoking. Attempts to lose and gain weight and bullying were associated with elevated odds of lifetime smoking across all sexual orientation groups. Bullying was associated with elevated odds of vaping among heterosexual, bisexual, and adolescent females not sure of their sexual orientation. Weight concern associations with vaping differed by sexual orientation; for example, attempts to gain weight were associated with elevated odds of vaping among all subgroups except lesbian adolescent females.

**Conclusion:**

Lesbian, bisexual, and adolescent females not sure of their sexual orientation were more likely to engage in smoking and vaping than their heterosexual peers. Disaggregating sexual minority subgroups revealed nuanced risk profiles for vaping, particularly for bisexual females, highlighting the importance of tailored prevention efforts to address sexual orientation disparities. Findings underscore the need for school-based interventions targeting weight concerns and bullying to address cigarette use and vaping.

## 1. Introduction

Tobacco use is the leading cause of preventable disease, disability, and death.^1^ Nearly all tobacco use begins in adolescence, with 9 out of 10 cigarette smokers trying their first cigarette by age 18.^2^ According to nationally representative data from the 2024 National Youth Tobacco Survey, vaping (i.e., use of e-cigarettes)—the most common form of nicotine use among youth in the United States (US)–decreased among adolescents, demonstrating the positive impact of tobacco regulatory efforts and public health prevention targeting youth.^3^ However, vaping remains high among young adults; according to 2023 data from the Monitoring the Future Study, 25.3% of young adults ages 19–30 vaped nicotine in the past 12 months, and 18.7% vaped nicotine in the past 30 days.^4^ Furthermore, because vaping is associated with use of combustible tobacco and other substance use (e.g., cannabis),^5,6^ more research is needed to inform early prevention of vaping, including research to address persistent disparities in all forms of tobacco and nicotine use. Despite declines in tobacco and nicotine use in the general population,^4^ lesbian, gay, bisexual, and other sexual minority individuals (i.e., those who do not identify as heterosexual) generally report elevated use relative to their heterosexual peers.^7,8^

Tobacco and nicotine use is particularly elevated among sexual minority females compared to heterosexual females. Analyzing data from 2015 to 2017 National Survey on Drug Use and Health, researchers found that among women over 18 years old, 17.1% of heterosexual women reported that they used cigarettes in the past month, compared to 28.1% of lesbians and 36.2% of bisexual women.^9^ Regarding vaping, using data from six major US metropolitan statistical areas, Romm and colleagues found that among females over 18 years old, 34.5% of heterosexual females, 42.9% of lesbian, and 57.5% of bisexual females reported vaping in the past 6 months.^8^ Sexual orientation health disparities in tobacco and nicotine use begin to emerge in adolescence.^10–12^ In research among adolescents in the Kentucky Youth Risk Behavior Survey, among LGB girls between 12 and 19 years old, 13.3% report cigarette use and 30.2% reported vaping in the past 30 days; these rates were higher than those reported by their heterosexual adolescent female peers, among who 6.6% reported cigarette use and 24.9% reported vaping.^13^

Identifying risk factors for tobacco and nicotine use among sexual minority females is a priority for prevention efforts. Prevailing theoretical frameworks that interpret sexual orientation disparities in tobacco and nicotine use have examined how minority stress (e.g., victimization, coping with stigma) contributes to elevated risk for smoking and other forms of substance use among sexual minority youth and adults.^14–16^ Adolescent girls, in general, may engage in smoking behaviors as a method of coping with stress and anxiety.^17^ Differential reports of stressors such as bullying (i.e., elevated rates among sexual minority females relative to heterosexual females) could contribute to observed differences in smoking among heterosexual and sexual minority adolescent females.^18^ For example, prior research has found that bisexual women are more likely than other sexual minority women to smoke due to particularly elevated experiences of marginalization.^19^

Research has also suggested that adolescent girls may engage in tobacco and nicotine use to prevent weight gain,^20^ as smoking is perceived to regulate appetite and suppress weight gain.^21,22^ Among adolescent females overall, cigarette use and vaping have been found to be strongly associated with intentions to lose weight.^23,24^ Sexual orientation subgroup differences in weight concern, particularly as they relate to tobacco and nicotine use behaviors, have not been sufficiently researched and merit closer attention. Although earlier research suggested that sexual minority women may report greater body satisfaction than heterosexual women,^24^ other research indicates that sexual minority women are also subjected to weight stigma and pressure to conform to thin appearance ideals.^25,26^ Furthermore, in studies adjusting for body weight, sexual orientation differences in body dissatisfaction and weight concern are less disparate or nonsignificant.^26,27^ Prior evidence indicates that weight concern may uniquely contribute to risk for tobacco and nicotine use above and beyond bullying; in one prior large-scale study of grade 9 to 12 students in Canada, weight loss attempts (a component of weight concern) and bullying were both significantly associated with increased cigarette use among adolescent girls, but this study did not examine differences by sexual orientation.^28^

More research is needed on how experiences of weight concern and bullying uniquely contribute to cigarette use and vaping among heterosexual and sexual minority adolescent females. The current study examined associations between weight concern (i.e., attempts to change weight, weight perceptions), exposure to bullying, and cigarette use behaviors (i.e., ever smoking in one’s lifetime, age of onset, current smoking) and vaping among heterosexual and sexual minority female adolescents. It was expected that desiring to lose weight would be associated with worse cigarette use and vaping outcomes. Consistent with overall sexual orientation disparities in cigarette use and vaping, it was expected that the association between desired weight loss and cigarette use and vaping outcomes would be stronger in sexual minority females than among heterosexual females. The current study also assessed the effect of bullying at school to explore whether exposure to bullying victimization (an indicator of minority stress exposure) further contributed to cigarette use and vaping among heterosexual and sexual minority female adolescents apart from weight concern.

## 2. Methods

### 2.1. Data source and measures

The Youth Risk Behavior Survey (YRBS) is a biennial national survey that has been conducted by the Centers for Disease Control and Prevention (CDC) since 1991 to collect health data on students in grades 9–12.^29^ The YRBS monitors priority health related behaviors among youth.^30^ For this study, we used data from local versions of the YRBS, which are administered on a state, large urban school district, or county level by departments of education or health; in this implementation, jurisdictions use a two-stage cluster sample design to identify a sample of students.^29^ In the first stage, schools are selected with a probability proportional to their enrollment; in the second stage, classes of a required subject or during a required period are randomly selected, and all students within these classes are eligible to participate. A new sample is selected in this manner each year that the survey is administered; the same students are not followed over time. Table 1 provides a detailed description of the variables used in the analysis.

### 2.2. Analytic sample

**Fig. 1.**
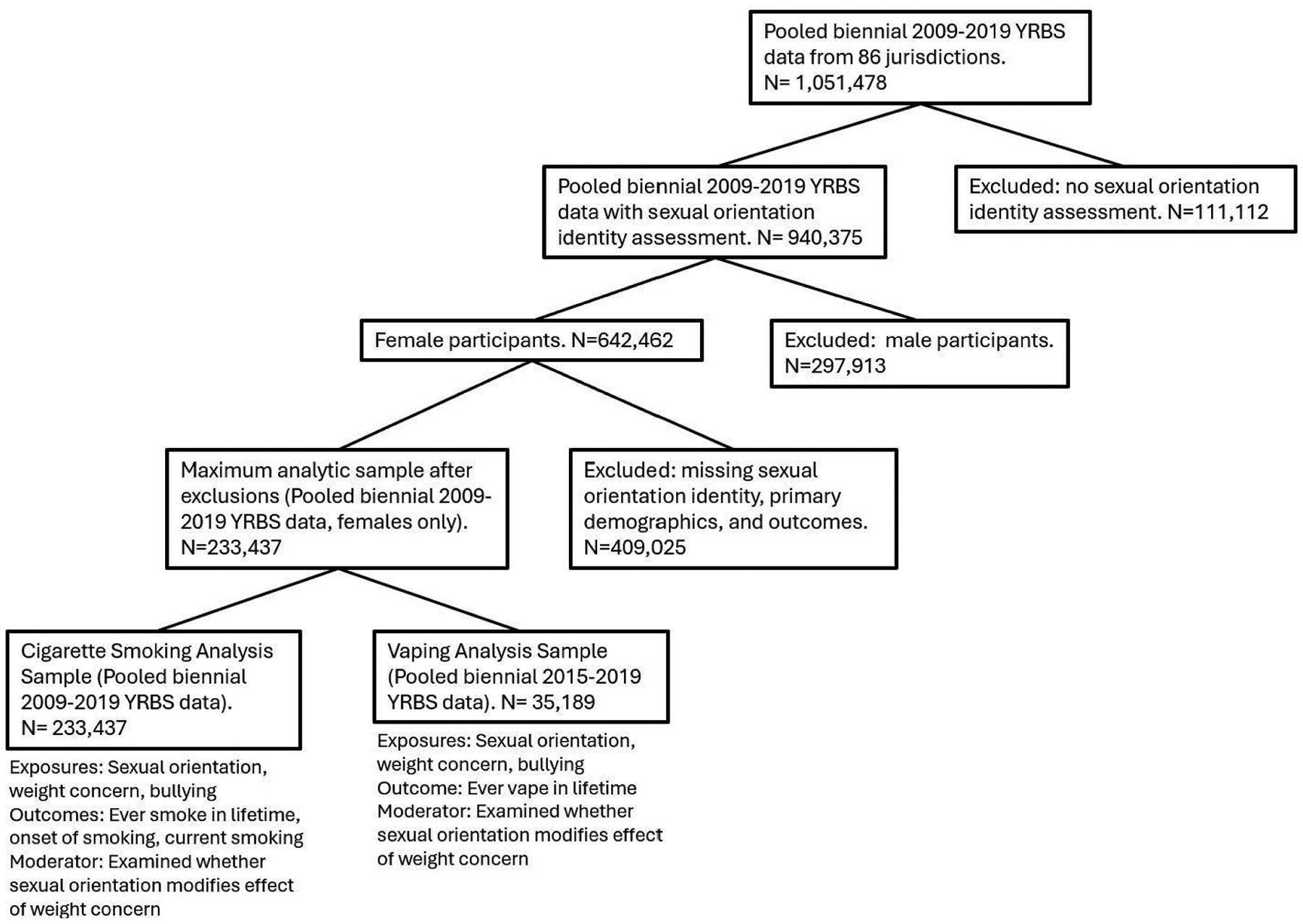
Flowchart depicting the two analytic samples derived from the 2009–2019 pooled, biennial Youth Risk Behavior Survey (YRBS) dataset and the exposures, outcomes, and moderators examined in cross-sectional, multivariable logistic regression models. Both sets of analyses included the covariates of body mass index (BMI) percentile/BMI, past 30-day binge drinking, past-year depressive distress, self-reported race/ethnicity, and age.

Local YRBS data were pooled across multiple jurisdictions and years (biennially from 2009 to 2019). The entire dataset consists of 86 jurisdictions across 6 time points, and 1,051,487 students. There were 699 jurisdiction-years (distinct surveys administered by a particular jurisdiction in a specific year) that assessed sexual identity (940,375 students). The present analysis uses data from female students only (n = 642,462). Students were excluded if they were missing sexual identity, any of the primary demographic variables of interest, or all outcomes (sexual identity: 30.75%; race/ethnicity: 2.40% and age 0.16%; tobacco use: 3.21%; not mutually exclusive) resulting in a maximum analytic sample size of 233,437 students (Fig. 1).

### 2.3. Statistical analysis

All data cleaning and recoding was conducted in SAS Version 9.4 (SAS Institute Cary, NC). Analyses were carried out using SAS-Callable SUDAAN Version 11.0.1 (RTI International, Research Triangle Park, NC) to appropriately weight estimates and to account for survey year and the complex sampling design of the YRBS. The YRBS data weights adjusts for student non-response and distribution of students by grade, sex, and race/ethnicity in each jurisdiction.^30^

First, descriptive statistics were calculated for smoking, weight concern, bullying, and covariates by sexual orientation identity in the pooled 2009–2019 dataset (Table 2). Second, to analyze cigarette use outcomes in the full dataset (n = 233,437; Fig. 1), multivariable logistic models were used to estimate odds for each of the three cigarette use outcomes: lifetime smoking, age of first smoke, current smoking. Models estimated the odds for each of the outcomes regressed on sexual orientation identity, the weight concern variables, bullying, and covariates (age; race/ethnicity; BMI; binge drinking; included if p < 0.05). Models tested for interactions between weight concern x sexual orientation identity to examine potential effect measure modification of weight concern change efforts by sexual orientation identity. Finally, the same multivariable logistic regression approach was used to examine vaping, but because vaping was only assessed in the 2015, 2017, and 2019 surveys, the dataset was restricted to those years for this analysis (n = 35,189; Fig. 1).

## 3. Results

### 3.1. Preliminary analyses

Table 2 displays descriptive statistics for key variables (weight concern, bullying) and the covariates by sexual orientation subgroup. Analyses for interactions found no significant interactions for weight change efforts or weight perception x sexual orientation identity and the cigarette use variables, thus indicating that the associations between weight change efforts and weight perception and cigarette use did not differ by sexual orientation. However, a significant weight change effort x sexual orientation identity interaction was detected for vaping (p = 0.0147), thus multivariable analyses for vaping were subsequently stratified by sexual orientation subgroup. All multivariable models (Tables 3 and 4) adjust for the significant covariates of binge drinking, depressive distress, BMI, race/ethnicity, and age.

### 3.2. Multivariable models

#### - Lifetime smoking

Lesbian, bisexual, and not sure adolescent females (i.e., participants who reported “not sure” for sexual orientation identity) reported elevated odds of ever smoking in their lifetime relative to heterosexual adolescent females (Table 3). Adolescent females who were attempting to lose weight (adjusted odds ratios [AOR] = 1.44, 95% confidence interval [CI] = 1.12, 1.85) and those attempting to gain weight (AOR = 1.48, 95% CI = 1.05, 2.08) had higher odds of ever smoking in their lifetime relative to participants who were not trying to do anything about their weight. Participants who reported experiencing bullying reported 1.41 times the odds of ever smoking in their lifetime relative to those who did not report bullying at school in the past year (95% CI = 1.14, 1.73).

#### - Age of initiation for smoking behavior

Bisexual adolescent females reported higher odds (AOR = 1.57, 95% CI = 1.08, 2.28) of starting to smoke before age 13 years old relative to heterosexual adolescent females (Table 3). Weight concern and bullying were not associated with age of initiation for smoking behavior.

#### - Current smoking

Lesbian (AOR = 3.92, 95% CI = 2.07, 7.42) and bisexual (AOR = 3.39, 95% CI = 2.39, 4.81) adolescent females had higher odds relative to heterosexual females of reporting being current smokers (Table 3). Weight concern and bullying were not associated with current smoking behavior.

#### - Vaping

Sexual orientation-stratified models revealed differential associations between weight concern and bullying variables and vaping by sexual orientation subgroup (Table 4). Among heterosexual adolescent females, those reporting attempts to lose weight (AOR = 1.34, 95% CI = 1.17, 1.53) or attempts to gain weight (AOR = 1.48, 95% CI = 1.21, 1.81) had elevated odds of vaping compared to heterosexual adolescent females who were not trying to do anything about their weight. Heterosexual adolescent females who reported experiencing bullying reported higher odds of vaping (AOR = 1.40, 95% CI = 1.25, 1.58) relative to those who reported no bullying.

Weight concern variables and bullying were not associated with vaping among lesbian adolescent females. Among bisexual adolescent females, attempts to lose weight (AOR = 1.45, 95% CI = 1.03, 2.05) or gain weight (AOR = 2.20, 95% CI = 1.33, 3.62) were associated with elevated odds of vaping relative to not trying to do anything about weight. Bisexual adolescent females who reported experiencing bullying at school reported higher odds (AOR = 1.48, 95% CI = 1.18, 1.85) of vaping relative to bisexual adolescent females who did not report experiencing bullying at school. Not sure adolescent females who reported attempting to gain weight had higher odds of vaping (AOR = 1.99, 95% CI = 1.06, 3.73) compared to those who were not trying to do anything about their weight. In addition, not sure adolescent females who experienced bullying at school had higher odds (AOR = 2.02, 95% CI = 1.41, 2.90) of vaping relative to those who did not experience bullying at school.

## 4. Discussion

Consistent with prior research,^10,11^ this study found that lesbian and bisexual adolescent females and not sure adolescent females were more likely to report ever smoking in their lifetime compared to heterosexual adolescent females. Lesbian and bisexual adolescent females had higher odds of being current smokers relative to heterosexual adolescent females, and bisexual females had higher odds of smoking prior to age 13 compared to heterosexual females. These findings support prior research on sexual orientation disparities in cigarette use and are novel in that they demonstrate that these disparities persist even after accounting for weight concern, bullying, and known covariates for cigarette use (e.g., binge drinking, depressive distress). The findings from this study underscore the associations between experiences of school-based bullying and elevated odds of cigarette use and vaping among adolescent females of all sexual orientations. In addition, weight loss and gain attempts were associated with lifetime smoking for adolescent females of all sexual orientations, and weight loss and gain attempts were associated with vaping among heterosexual and bisexual adolescent females.

Some literature has shown that bisexual women are more likely than other sexual minority women to smoke due to increased experiences of marginalization, exclusion, and conflict from experiences of not belonging or being accepted in straight, lesbian, or gay communities.^19^ For example, bisexual women smoke cigarettes and vape at younger ages, more intensely, and with greater nicotine dependence than lesbian or heterosexual women.^8,19^ This may be in part due to unique stressors such as internalized biphobia and alienation from straight and gay/-lesbian communities.^19^ In the current study, bisexual adolescent females had higher odds than heterosexual females of smoking prior to age 13 and being current smokers. Furthermore, in sexual orientation stratified models, weight change efforts and experiences with school-based bullying were associated with elevated odds of vaping among bisexual adolescent females.

Contrary to expectations and prior research that only attempts to lose weight would be associated with cigarette use and vaping, the current study found that attempts to gain weight were also associated with elevated odds of adolescent females ever smoking in their lifetime, and in vaping behavior among heterosexual, bisexual, and not sure adolescent females. Prior research has found that vaping is associated with weight gain attempts in adolescent boys,^23^ and that substance use behaviors (i.e., alcohol use, binge-drinking, cigarette smoking, cannabis use) are more prevalent among adults who are attempting to gain weight relative to those who are not attempting to do anything to their weight.^33^ Adolescent females who are attempting to gain weight may be similar to adolescent females who are attempting to lose weight in that both groups may experience weight concern and body dissatisfaction. Cigarette use and vaping could be used to mitigate stress related to weight concern, body dissatisfaction and weight stigma. Alternatively, alongside attempts to gain weight, some adolescent females may engage in cigarette use and vaping as an attempt to be socially accepted as mature by their peers.^34^ It is also possible that cigarette smoking and weight gain attempts co-occur due to a shared underlying trans-diagnostic mechanism (i.e., impulsivity and/or reward seeking), or may co-occur as a means of suppressing overall appetite while pursuing specific appearance-related goals (e.g., lean muscularity).^35^ Given the relative dearth of research on weight gain attempts within body image research,^33^ particularly among adolescent girls and women, future research should further explicate the association between weight gain attempts and smoking and vaping behaviors among adolescent females of all sexual orientations.

Although analyses found that the effect of weight change efforts and vaping differed by sexual orientation, there were no associations between weight change efforts, weight perceptions, bullying, and vaping among lesbian adolescent females. The findings highlight that sexual minority females should not be treated as a monolith. In sexual orientation-stratified models, attempts to lose or gain weight were associated with elevated odds of vaping among both heterosexual and bisexual adolescent females, and attempts to gain weight were associated with elevated odds of vaping among heterosexual, bisexual, and not sure adolescent females. Bullying was associated with elevated odds of vaping among heterosexual, bisexual, and not sure adolescent females. Taken together, exposure to school-based bullying, regardless of sexual orientation identity, contributes to risk for cigarette use behaviors and/or vaping among adolescent females of all sexual orientations. Other research has found robust associations between exposure to bullying and cigarette smoking and vaping among adolescent females,^36,37^ further corroborating the need for preventive interventions that promote school safety. The current study only assessed exposure to school-based bullying. However, some research has identified differential associations between bullying perpetration, victimization, or being a bully-victim and cigarette use and vaping behavior.^37^

Regarding limitations, data are limited to 2009–2019, and are cross-sectional, limiting ascertainment of directionality of associations. Other research has delineated complex, longitudinal associations between victimization and weight change,^38^ or BMI and cigarette use.^39^ While the analyses are unique in their examination of weight concern, bullying, and smoking behaviors among adolescent females of different sexual orientation groups, the prevalence estimates must be evaluated against epidemiologic data post-2019. However, more recent large-scale epidemiologic surveillance of adolescent samples (e.g., 2023 National Youth Tobacco Survey data) continue to report elevated tobacco use among sexual minority youth compared to their heterosexual peers.^40^ In addition, we note that other research has found substantial changes in adolescent and young adult smoking and vaping behavior due to the COVID-19 pandemic,^41,42^ thus suggesting that 2021 YRBS data (for example) should be examined separately from the pooled data. Although measures in the YRBS have been developed for large-scale epidemiologic surveillance with adolescents, the measures are based on single-item assessments and self-report. Recent research across cycles of the YRBS has identified gender-related changes in the proportion of youth who identify as being “unsure” of their sexual orientation identity, providing evidence that this may be a heterogenous subgroup consisting of youth who are unsure of their sexual orientation and youth who are not sure what the question is asking about.^43^ Findings in this study regarding not sure adolescent females should be interpreted with some caution, or examined more closely with additional questions that enhance reliability of assessment of sexual orientation identity.^43^ The school-based bullying question only asked about victimization and not perpetration; some research has identified unique associations between victimization, perpetration, and smoking.^44^ The YRBS does not capture the experiences of youth who are absent from school on the day of assessment. Prior research has indicated that sexual minority youth are more likely than their heterosexual peers to experience absenteeism and truancy, largely because of school-based victimization.^45^

Despite these limitations, the current study exhibited several strengths, and the findings have implications for future research and practice. The study pooled survey data across multiple cycles of the YRBS to ensure adequate power to investigate the research question, while also disaggregating sexual minority subgroups for multivariable and stratified analyses. Weight loss and gain attempts were associated with lifetime smoking for adolescent females of all sexual orientations and were associated with vaping among heterosexual and bisexual adolescent females, but not among lesbian and not sure adolescent females. While the findings indicate that weight concern may not affect lifetime cigarette use differentially by sexual orientation, more research is needed to understand the differential contributions of weight concern to vaping risk by sexual orientation. Overall, the findings indicate that weight concerns in either direction (towards weight loss or gain) were generally associated with elevated odds of lifetime smoking and vaping among adolescent females of diverse sexual orientation identities. Because tobacco and vaping products have a long history of being marketed for weight control,^46^ the findings of the current study further emphasize the need for regulations around the marketing and promotion of cigarettes and vaping products with weight control claims. School-based interventions targeting weight concerns in general could potentially mitigate the impact of different forms of weight concern on smoking risk. The findings also underscore the negative impact of school-based bullying on cigarette use and vaping among adolescent females of all sexual orientations. Efforts to ensure that school spaces are safe may help to mitigate cigarette use and vaping behavior and other upstream determinants and protective factors for such behaviors (e.g., mental health, school engagement and academic achievement).^47^

## Figures and Tables

**Table 1:** Operationalization of key measures.

Measure	Question	Response Options/Recoding
Exposures		
Sexual Orientation Identity	Which of the following best describes you?	Heterosexual (Straight) (referent) Gay or lesbian Bisexual Not Sure
Weight Concern: *Weight change effort*	Which of the following are you trying to do about your weight?	Lose weight Gain weight Stay the same weight I am not trying to do anything about my weight (referent)
Weight Concern: *Weight perception*	How do you describe your weight?	Very underweight Slightly underweight About the right weight Slightly overweight Very overweight Response options were recoded into three categories: “Underweight”, “About the right weight” (referent), “Overweight”
Bullying	During the past 12 months, have you ever been bullied on school property?	Yes No
**Outcomes**		
Tobacco Use: *Lifetime smoking*	Have you ever tried cigarette smoking, even one or two puffs?	Yes No
Tobacco Use: *Age of first cigarette*	How old were you when you smoked a whole cigarette for the first time?	Responses were coded to reflect those who began smoking when they were “younger than 13” and those who began smoking at “13” years or older”, per the CDC dichotomization used in the 2015 YRBS Combined Data Users Guide^31^
Tobacco Use: *Current cigarette use*	During the past 30 days, on how many days did you smoke cigarettes?	Response options ranged from 0 to 29 days. Based on the distribution of response, responses were collapsed and dichotomized as “Any use” or “No use”.
Vaping	Have you ever used an electronic vapor product? *(2015, 2017, 2019 years)*	Yes No
**Covariates**		
Year	The year the survey was completed	The year the survey was completed
Age	How old are you?	Seven response options ranged from 12 years old or younger to 18 years old or older. Three of the items for age were collapsed into a single category resulting in an age variable with five categories: “14 or younger”; “15 years old”; “16 years old”; “17 years old”; and “18 and older” (referent)
Race/Ethnicity	Are you Hispanic or Latino? What is your race? (Select one or more responses)	Yes, No American Indian or Alaska Native; Asian; Black or African American; Native Hawaiian or Other Pacific Islander; White Using CDC’s classification, variables were combined into racial/ethnic groups: “American Indian/Alaska Native”; “Asian”; “Black or African American”; “Hispanic/Latino”; “Native Hawaiian/Pacific Islander”; “White”; “Multiple Races (Non-Hispanic)
Body Mass Index	Self-reported height and weight	BMI was calculated from self-reported height and weight and categorized into sex and age-specific percentile ranges corresponding to underweight, healthy weight, overweight, and obese for participants younger than age 18, and similar weight status category ranges based on raw BMI scores for adult BMI participants age 18 years and older^32^
Binge Drinking	During the past 30 days, on how many days did you have 5 or more drinks of alcohol in a row, that is, within a couple of hours?	Response options range from 0 to 20+ days, and were coded into three categories: “0 days” (referent), “1–2 days”, and “3+ days”.
Depressive Distress	During the past 12 months, did you ever feel so sad or hopeless almost every day for two weeks or more in a row that you stopped doing some usual activities?	Yes No

**Table 2:** Sample descriptive statistics for key variables by sexual orientation subgroup among adolescent females in the 2009–2019 pooled youth risk behavior surveys (n = 233,437).

	Heterosexual N = 192,316			Lesbian N = 5518			Bisexual N = 24,483			Not Sure N = 11,120		
	n	%	95% CI	n	%	95% CI	n	%	95% CI	n	%	95% CI
**Cigarette Use**												
*Lifetime Smoking*												
Yes	12,067	18.87	18.70, 19.04	771	36.52	35.25, 37.79	3739	31.55	30.97, 32.13	974	21.51	20.75, 22.27
No	52,956	81.13	80.96, 21.30	1484	63.48	62.21, 64.75	6921	68.45	67.87, 69.03	3434	78.49	77.73, 79.25
*Age of First Smoke*												
Younger than 13	3565	30.25	30.04, 30.46	345	37.68	36.40, 38.96	1378	37.56	36.95, 38.17	375	38.92	38.01, 39.83
13 or older	8502	69.75	69.54, 69.96	426	62.32	61.04, 63.60	2361	62.44	61.83, 63.05	599	61.08	60.17, 61.99
*Currently Smoke (in the Past 30 days)*												
Yes	12,340	6.51	6.40, 6.62	887	16.81	15.82, 17.80	4106	17.22	16.75, 17.69	1021	8.96	8.43, 9.49
No	170,025	93.49	93.38, 93.60	4014	83.19	82.20, 84.18	18,403	82.78	82.31, 83.25	9308	91.04	90.51, 91.57
Electronic Vapor Products Use												
Yes	38,264	42.59	42.39, 42.81	1693	57.04	55.73, 58.35	7956	52.31	51.68, 52.94	2172	32.90	32.03, 33.77
No	58,598	57.41	57.19, 57.63	1522	42.96	41.65, 44.27	6873	47.69	47.06, 48.32	4083	67.10	66.23, 67.97
**Weight Concern**												
*Weight Change Effort*												
Lose weight	84,928	59.27	59.05, 59.49	2179	56.90	55.59, 58.21	11,156	61.71	61.10, 62.32	4312	54.93	54.01, 55.85
Gain weight	11,839	8.43	8.31, 8.55	565	11.45	10.61, 12.29	1778	9.39	9.02, 9.76	784	9.69	9.14, 10.24
Stay the same weight	26,044	16.90	16.73, 17.06	576	15.44	14.49, 16.39	2063	12.82	12.40, 13.24	1019	12.86	12.24, 13.48
Not trying to do anything about weight	23,931	15.40	15.24, 15.56	686	16.21	15.24, 17.18	2903	16.08	15.62, 16.54	1737	22.53	21.75, 23.31
*Weight Perception*												
Underweight	17,347	12.06	11.91, 12.21	836	19.24	18.20, 20.28	2481	14.99	14.54, 15.44	1298	15.15	14.48, 15.82
About the right weight	81,025	54.08	53.86, 54.30	1657	39.07	37.78, 40.36	7190	38.85	38.24, 39.46	3281	42.24	41.32, 43.16
Overweight	49,427	33.86	33.65, 34.07	1561	41.69	40.40, 43.00	8282	46.16	45.54, 46.78	3343	42.61	41.69, 43.53
**Bullying**												
Yes	28,670	19.64	19.46, 19.82	1191	30.10	28.89, 31.31	6158	31.48	30.90, 32.06	2329	29.90	29.05, 30.75
No	129,147	80.36	80.18, 80.53	3386	69.90	68.69, 71.11	13,664	68.52	67.94, 69.10	6769	70.10	69.25, 70.95
***COVARIATES***												
*Age*												
14 or younger	27,857	13.33	13.18, 13.48	682	11.01	10.18, 11.84	3276	12.42	12.01, 12.83	2022	18.61	17.89, 19.33
15 years old	50,467	25.20	25.00, 25.39	1317	24.83	23.69, 25.97	6494	24.15	23.61, 24.69	3110	29.08	28.24, 29.92
16 years old	49,625	25.58	25.38, 25.78	1455	24.07	22.94, 25.20	6698	26.91	26.35, 27.47	2772	24.60	23.80, 25.40
17 years old	44,516	23.57	23.38, 23.76	1325	26.20	25.04, 27.36	5522	23.80	23.27, 24.33	2071	17.58	16.87, 18.29
18 or older	19,851	13.32	13.17, 13.47	739	13.90	12.99, 14.81	2493	12.72	12.30, 13.14	1145	10.13	9.57, 10.69
*Race/Ethnicity*												
American Indian/Alaska Native	3072	0.89	0.85, 0.93	100	0.87	0.62, 1.12	597	1.26	1.12, 1.40	231	1.27	1.06, 1.48
Asian	11,982	5.70	5.60, 5.80	176	2.24	1.85, 2.63	885	5.20	4.92, 5.48	1074	8.63	8.11, 9.15
Black or African American	25,412	13.68	13.52, 13.83	1167	22.84	21.73, 23.95	3517	15.43	14.98, 15.88	1543	13.54	12.90, 14.18
Hispanic/Latino	46,086	29.02	28.81, 29.22	1603	29.36	28.16, 30.56	7119	32.04	31.46, 32.62	31.45	30.79	29.93, 31.65
Native Hawaiian/PI	2968	0.60	0.57, 0.63	95	0.64	0.43, 0.85	293	0.44	0.36, 0.52	169	1.32	1.11, 1.53
White	93,660	46.52	46.30, 46.74	2016	39.73	38.44, 41.02	10,298	40.57	39.95, 41.19	4314	39.16	38.25, 40.07
Multiple Races (Non-Hispanic)	9136	3.59	3.51, 3.67	361	4.32	3.78, 4.86	1774	5.07	4.80, 5.34	664	5.29	4.87, 5.71
*BMI Percentile/BMI*												
< 5th Percentile/BMI <18.5	17,681	8.16	8.04, 8.28	708	10.43	9.62, 11.24	2407	8.84	8.48, 9.20	1778	13.73	13.09, 14.37
≥ 5th Percentile, <85th Percentile/BMI 18.5–24.9	131,367	68.76	68.55, 68.97	3034	53.94	52.62, 55.26	13,698	57.34	56.72, 57.96	6202	57.37	56.45, 58.29
≥ 85th Percentile, <95th Percentile/BMI 25–29.9	27,591	14.83	14.67, 14.99	959	20.49	19.43, 21.55	4421	17.12	16.65, 17.59	1714	15.43	14.76, 16.10
≥ 95th Percentile/BMI ≥30	15,677	8.25	8.13, 8.37	817	15.15	14.20, 16.10	3966	16.70	16.23, 17.17	1426	13.47	12.84, 14.10
*Binge Drinking*												
0 days	63,175	87.14	86.99, 87.29	2136	84.09	83.12, 85.06	9727	83.99	83.53, 84.45	4733	90.71	90.17, 91.25
1–2 days	6162	8.63	8.50. 8.76	251	12.13	11.27, 12.99	1277	11.42	11.02, 11.82	350	6.03	5.59, 6.47
3+ days	2792	4.23	4.14, 4.32	126	3.78	3.28, 4.28	629	4.59	4.33, 4.85	196	3.26	2.92, 3.59
*Past-Year Depressive Distress*												
Yes	61,933	35.08	34.87, 35.29	2992	58.80	57.50, 60.10	15655	69.48	68.90, 70.06	5372	53.06	52.13, 53.99
No	128,735	64.92	64.71, 65.13	2326	41.20	39.90, 42.50	8497	30.52	29.94, 31.10	5560	46.94	46.01, 47.97

Note: 95% CI = 95% Confidence interval for the proportion; BMI= Body mass index.

**Table 3:** Multivariable logistic regression models examining associations between sexual orientation, weight concern, bullying, and smoking behaviors among adolescent females in the 2009–2019 youth risk behavior surveys (n = 233,437).

	Lifetime Smoking			First Smoke Prior to Age 13 Years			Current Smoking		
	AOR	95% CI	p-value	AOR	95% CI	p-value	AOR	95% CI	p-value
*Sexual Orientation Identity*									
Heterosexual	REF	–	–	REF	–	–	REF	–	–
Lesbian	**2.65**	**(1.56, 4.51)**	**0.0003**	2.17	(0.94, 5.02)	0.0706	**3.92**	**(2.07, 7.42)**	**0.0000**
Bisexual	**3.08**	**(2.29, 4.13)**	**0.0000**	**1.57**	**(1.08, 2.28)**	**0.0172**	**3.39**	**(2.39, 4.81)**	**0.0000**
Not Sure	**1.77**	**(1.12, 2.78)**	**0.0138**	1.25	(0.66, 2.40)	0.4929	1.74	(0.92, 3.29)	0.0885
*Weight Concern*									
*Weight Change Effort*									
Lose Weight	**1.44**	**(1.12, 1.85)**	**0.0043**	1.40	(0.89, 2.22)	0.1475	1.34	(0.95, 1.88)	0.0965
Gain Weight	**1.48**	**(1.05, 2.08)**	**0.0258**	1.82	(0.97, 3.43)	0.0625	1.37	(0.85, 2.21)	0.1969
Stay the same Weight	1.13	(0.82, 1.55)	0.4593	1.30	(0.75, 2.26)	0.3537	0.84	(0.51, 1.39)	0.4934
Not trying to do anything about Weight	REF	–	–	REF	–	–	REF	–	–
*Weight Perception*									
Underweight	0.95	(0.73, 1.23)	0.6744	1.26	(0.74, 2.13)	0.3945	0.93	(0.66, 1.31)	0.6751
About the Right Weight	REF	–	–	REF	–	–	REF	–	–
Overweight	0.91	(0.75, 1.11)	0.3714	1.00	(0.73, 1.38)	0.9813	0.80	(0.60, 1.06)	0.1248
*Bullying*									
Yes	**1.41**	**(1.14, 1.73)**	**0.0013**	1.18	(0.81, 1.71)	0.3884	1.26	(0.93, 1.70)	0.1334
No	REF	–	–	REF	–	–	REF	–	–
*COVARIATES*									
*Age*									
14 or Younger	**0.50**	**(0.37, 0.66)**	**0.0000**	1.93	**(1.07, 3.49)**	**0.0295**	**0.59**	**(0.42, 0.85)**	**0.0039**
15 years old	**0.51**	**(0.40, 0.64)**	**0.0000**	1.69	**(1.06, 2.68)**	**0.0278**	**0.60**	**(0.44, 0.83)**	**0.0017**
16 years old	**0.59**	**(0.46, 0.75)**	**0.0000**	1.40	(0.85, 2.31)	0.1811	**0.64**	**(0.46, 0.88)**	**0.0062**
17 years old	**0.75**	**(0.60, 0.93)**	**0.0101**	1.08	(0.69, 1.68)	0.7507	**0.75**	**(0.57, 0.98)**	**0.0323**
18 or older	REF	–	–	REF	–	–	REF	–	–
*Race/Ethnicity*									
American Indian/Alaska Native	0.59	(0.19, 1.83)	0.3578	2.00	(0.48, 8.33)	0.3414	1.13	(0.41, 3.12)	0.8105
Asian	**0.57**	**(0.39, 0.84)**	**0.0044**	2.51	**(1.46, 4.32)**	**0.0009**	**0.07**	**(0.02, 0.23)**	**0.0000**
Black or African American	**0.53**	**(0.41, 0.68)**	**0.0000**	1.63	(0.96, 2.78)	0.0729	**0.29**	**(0.19, 0.43)**	**0.0000**
Hispanic/Latino	0.99	(0.83, 1.19)	0.9426	0.97	(0.68, 1.40)	0.8849	**0.60**	**(0.46, 0.79)**	**0.0003**
Native Hawaiian/PI	**0.34**	**(0.12, 0.93)**	**0.0365**	2.79	(0.53, 14.78)	0.2283	0.32	(0.07, 1.47)	0.1431
White	REF	–	–	REF	–	–	REF	–	–
Multiple Races (Non-Hispanic)	0.99	(0.69, 1.43)	0.9669	1.53	(0.86, 2.71)	0.1449	1.51	(0.98, 2.34)	0.0645
*BMI Percentile/BMI*									
< 5th Percentile/BMI <18.5	0.97	(0.75, 1.25)	0.8253	1.01	(0.57, 1.78)	0.9728	1.21	(0.85, 1.73)	0.2980
≥ 5th Percentile, <85th Percentile/BMI 18.5–24.9	REF	–	–	REF	–	–	REF	–	–
≥ 85th Percentile, <95th Percentile/BMI 25–29.9	1.19	(0.94, 1.51)	0.1502	1.68	**(1.11, 2.54)**	**0.0144**	**1.46**	**(1.01, 2.12)**	**0.0447**
≥ 95th Percentile/BMI ≥30	**1.44**	**(1.08, 1.93)**	**0.0125**	2.02	**(1.25, 3.28)**	**0.0043**	**1.58**	**(1.01, 2.49)**	**0.0466**
*Binge Drinking (past 30 days)*									
0 days	REF	–	–	REF	–	–	REF	–	–
1–2 days	**4.31**	**(3.53, 5.27)**	**0.0000**	1.03	(0.69, 1.53)	0.8906	**5.17**	**(4.05, 6.60)**	**0.0000**
3+ days	**9.06**	**(6.93, 11.86)**	**0.0000**	1.76	**(1.22, 2.55)**	**0.0026**	**13.59**	**(10.10, 18.28)**	**0.0000**
*Past-Year Depressive Distress*									
Yes	REF			REF			REF		
No	**0.53**	**(0.46, 0.61)**	**0.0000**	0.90	(0.69, 1.17)	0.4203	**0.53**	**(0.43, 0.66)**	**0.0000**

Note: AOR = Adjusted odds ratio; 95% CI = 95% Confidence interval for the AOR; REF= Referent; – = not estimated; BMI= Body mass index.

**Table 4:** Multivariable logistic regression models examining associations between weight concern, bullying, and vaping among heterosexual and sexual minority adolescent females in the 2015–2019 youth risk behavior surveys (n = 35,189).

	Heterosexual			Lesbian			Bisexual			Not Sure		
	AOR	95% CI	p-value	AOR	95% CI	p-value	AOR	95% CI	p-value	AOR	95% CI	p-value
*Weight Change Effort*												
Lose Weight	**1.34**	**1.17, 1.53**	**0.0000**	1.59	0.85, 2.98	0.1458	**1.45**	**1.03, 2.05**	**0.0349**	1.43	0.84, 2.46	0.1894
Gain Weight	**1.48**	**1.21, 1.81**	**0.0001**	0.78	0.35, 1.70	0.5248	**2.20**	**1.33, 3.62**	**0.0020**	**1.99**	**1.06, 3.73**	**0.0311**
Stay the same Weight	1.09	0.92, 1.29	0.3361	1.30	0.62, 2.72	0.4855	1.05	0.68, 1.62	0.8277	1.00	0.55, 1.83	0.9892
Not trying to do anything about	REF	–	–	REF	–	–	REF	–	–	REF	–	–
Weight												
***Weight Perception***												
Underweight	1.16	0.99, 1.35	0.0660	1.59	0.83, 3.02	0.1597	0.89	0.57, 1.39	0.6054	1.57	0.79, 3.15	0.1995
About the Right Weight	REF			REF			REF			REF		
Overweight	0.95	0.84, 1.06	0.3502	1.23	0.65, 2.35	0.5230	0.98	0.71, 1.35	0.8818	1.11	0.65, 1.92	0.7026
***Bullying***												
Yes	**1.40**	**1.25, 1.58**	**0.0000**	1.39	0.84, 2.30	0.1970	**1.48**	**1.18, 1.85**	**0.0006**	**2.02**	**1.41, 2.90**	**0.0001**
No	REF	–	–	REF	–	–	REF	–	–	REF	–	–
***COVARIATES***												
***Age***												
14 or Younger	**0.56**	**0.46, 0.67**	**0.0000**	0.51	0.23, 1.15	0.1038	**0.51**	**0.31, 0.83**	**0.0067**	0.89	0.44, 1.84	0.7611
15 years old	**0.70**	**0.61, 0.81**	**0.0000**	0.59	0.29, 1.18	0.1356	0.83	0.55, 1.24	0.3556	0.75	0.36, 1.55	0.4375
16 years old	0.94	0.81, 1.10	0.4388	0.91	0.43, 1.94	0.8091	0.86	0.55, 1.33	0.4893	1.07	0.54, 2.13	0.8425
17 years old	0.99	0.86, 1.14	0.9277	0.74	0.39, 1.40	0.3493	1.00	0.66, 1.50	0.9868	1.60	0.77, 3.35	0.2104
18 or older	REF	–	–	REF	–	–	REF	–	–	REF	–	–
***Race/Ethnicity***												
American Indian/Alaska Native	1.11	0.69, 1.80	0.6663	2.19	0.56, 8.53	0.2587	0.68	0.22, 2.14	0.5114	1.06	0.32, 3.49	0.9283
Asian	**0.44**	**0.35, 0.56**	**0.0000**	1.72	0.41, 7.30	0.4609	**0.34**	**0.18, 0.61**	**0.0004**	**0.31**	**0.14, 0.69**	**0.0045**
Black or African American	**0.72**	**0.63, 0.81**	**0.0000**	1.44	0.86, 2.39	0.1619	**0.69**	**0.53, 0.91**	**0.0094**	0.81	0.54, 1.22	0.3141
Hispanic/Latino	**0.88**	**0.78, 0.99**	**0.0364**	1.27	0.73, 2.22	0.4013	1.06	0.82, 1.38	0.6556	1.12	0.72, 1.73	0.6215
Native Hawaiian/PI	1.52	0.81, 2.83	0.1914	6.84	0.74, 62.76	0.0892	**0.24**	**0.07, 0.80**	**0.0201**	0.54	0.06, 4.78	0.5833
White	REF	–	–	REF	–	–	REF	–	–	REF	–	–
Multiple Races (Non-Hispanic)	0.95	0.76, 1.20	0.6765	0.79	0.33, 1.90	0.5964	1.13	0.78, 1.65	0.5200	1.98	0.91, 4.28	0.0838
***BMI Percentile/BMI***												
<5th *Percentile/BMI < 18.5*	**0.71**	**0.59, 0.87**	**0.0007**	0.63	0.25, 1.57	0.3203	1.47	0.92, 2.34	0.1081	0.75	0.41, 1.38	0.3567
>5th *Percentile,* <85th *Percentile/BMI 18.5–24.9*	REF	–	–	REF	–	–	REF	–	–	REF	–	–
> 85th *Percentile,* < 95th *Percentile/BMI 25–29.9*	1.03	0.91, 1.18	0.6147	1.16	0.66, 2.05	0.6096	1.15	0.85, 1.57	0.3688	0.94	0.62, 1.45	0.7951
> 95th *Percentile/BMI > 30*	1.04	0.86, 1.26	0.7001	1.46	0.73, 2.89	0.2831	1.14	0.78, 1.66	0.4995	0.91	0.53, 1.56	0.7316
***Binge Drinking (past 30 days)***												
0 days	REF	–	–	REF	–	–	REF	–	–	REF	–	–
1–2 days	**5.31**	**4.30, 6.57**	**0.0000**	**22.44**	**8.93, 56.39**	**0.0000**	**4.14**	**2.69, 6.39**	**0.0000**	**6.15**	**2.72, 13.90**	**0.0000**
3+ days	**12.50**	**8.23, 18.98**	**0.0000**	**9.39**	**2.47, 35.77**	**0.0010**	**13.32**	**7.08, 25.08**	**0.0000**	**11.14**	**2.52, 49.23**	**0.0015**
***Past-Year Depressive Distress***												
Yes	REF	–	–	REF	–	–	REF	–	–	REF	–	–
No	**0.52**	**0.48, 0.57**	**0.0000**	0.89	0.57, 1.38	0.5891	**0.66**	**0.53, 0.82**	**0.0002**	**0.53**	**0.36, 0.78**	**0.0012**

Note: AOR = Adjusted odds ratio; 95% CI = 95% Confidence interval for the AOR; REF= Referent; – = not estimated; BMI= Body mass index.

## Data Availability

Data underlying this article were accessed from the U.S. Centers for Disease Control and Prevention Youth Risk Behavior Surveillance System (YRBSS) (https://www.cdc.gov/yrbs/data/index.html).
